# “In Litero” Screening: Retrospective Evaluation of Clinical Evidence to Establish a Reference List of Human Chemical Respiratory Sensitizers

**DOI:** 10.3389/ftox.2022.916370

**Published:** 2022-07-15

**Authors:** Jessica Ponder, Ramya Rajagopal, Madhuri Singal, Nancy Baker, Grace Patlewicz, Erwin Roggen, Stella Cochrane, Kristie Sullivan

**Affiliations:** ^1^ Physicians Committee for Responsible Medicine, Washington, D.C., DC, United States; ^2^ SEAC, Unilever, Sharnbrook, United Kingdom; ^3^ AeroTox Consulting Services, LLC, Montvale, NJ, United States; ^4^ Leidos Contractor to the US EPA, Research Triangle Park, Durham, NC, United States; ^5^ US EPA, Research Triangle Park, Washington, NC, United States; ^6^ Danish 3R-Center, Glostrup, Denmark

**Keywords:** respiratory sensitization, respiratory sensitisation, adverse outcome pathway, chemical allergens, occupational asthma, allergic asthma, clinical reference list, new approach methodologies

## Abstract

Despite decades of investigation, test methods to identify respiratory sensitizers remain an unmet regulatory need. In order to support the evaluation of New Approach Methodologies in development, we sought to establish a reference set of low molecular weight respiratory sensitizers based on case reports of occupational asthma. In this context, we have developed an “in litero” approach to identify cases of low molecular weight chemical exposures leading to respiratory sensitization in clinical literature. We utilized the EPA-developed Abstract Sifter literature review tool to maximize the retrieval of publications relevant to respiratory effects in humans for each chemical in a list of chemicals suspected of inducing respiratory sensitization. The literature retrieved for each of these candidate chemicals was sifted to identify relevant case reports and studies, and then evaluated by applying defined selection criteria. Clinical diagnostic criteria were defined around exposure history, respiratory effects, and specific immune response to conclusively demonstrate occupational asthma as a result of sensitization, rather than irritation. This approach successfully identified 28 chemicals that can be considered as human respiratory sensitizers and used to evaluate the performance of NAMs as part of a weight of evidence approach to identify novel respiratory sensitizers. Further, these results have immediate implications for the development and refinement of predictive tools to distinguish between skin and respiratory sensitizers. A comparison of the protein binding mechanisms of our identified “in litero” clinical respiratory sensitizers shows that acylation is a prevalent protein binding mechanism, in contrast to Michael addition and Schiff base formation common to skin sensitizers. Overall, this approach provides an exemplary method to evaluate and apply human data as part of the weight of evidence when establishing reference chemical lists.

## 1 Introduction

Respiratory sensitization (RS) from exposure to low molecular weight (LMW) chemicals is a significant occupational health hazard in a number of industries, especially from the manufacture and use of wood, epoxy resins and paints, biocides, pharmaceuticals and other medical applications (Zacharisen, 2002) as well as consumer products in both occupational and consumer use settings (Maier et al., 2014). Both respiratory and dermal chemical allergies typically cause lifelong sensitivity to the sensitizing material(s), for which the only effective management is avoidance. RS is defined as airway hyper-responsiveness to a substance and is distinguished from skin sensitization by the symptoms rather than route of exposure, as it is hypothesized that RS may also be caused by dermal exposures (Kimber and Dearman, 2002). There are no regulatory-accepted *in vivo* test methods for this endpoint (Chary et al., 2018). However, progress in understanding the molecular mechanisms leading to the activation of an immune response to LMW chemicals has provided insight that can aid the development of New Approach Methodologies (NAMs) including *in silico* and *in vitro* approaches with the potential to reliably predict human responses to respiratory sensitizers (Kimber et al., 2011).

A key input in the development of a hazard characterization approach is the use of a high-confidence reference set of chemicals. The predictive capacity of NAMs and the resulting confidence in their implementation for regulatory testing, is inherently dependent on the quality of the chemical reference set used to evaluate the accuracy of the assays. In order to promote the development of the most human-relevant test methods for RS, we established a clinically driven approach to identifying low molecular weight chemicals that have been conclusively shown to cause RS in humans. Where acquiring controlled experimental human data is not feasible for hazard identification, as is the case for most toxicological endpoints, clinical literature provides significant utility for determining which chemicals have induced a given response in the most relevant setting. Herein we describe our “in litero” screening approach, utilizing a review of epidemiological reports of occupational asthma with attention to evidence of molecular mechanisms consistent with respiratory sensitization for each of the potential sensitizers investigated, resulting in the positive identification of 28 clinical respiratory sensitizers.

This approach was guided by a focus on the mechanisms outlined in the Adverse Outcome Pathway (AOP) for Respiratory Sensitization by LMW chemicals. AOPs describe the process of how an adverse outcome, such as sensitization, progresses from an initiating event after exposure to a stressor, such as an electrophile (Ankley et al., 2010). The AOP for RS^1^, under development by the Organisation for Economic Co-operation and Development (OECD) reflects our current understanding of the molecular initiating event and key events leading to the activation of effector T-cells which constitute the hallmark of an allergic response (Sullivan et al., 2017). The complete process of sensitization from chemical exposure has proven difficult to induce in mammals, especially for respiratory sensitizers (Chapman et al., 2014). Fortunately, earlier key events are highly amenable to modeling using NAMs. Low molecular weight chemicals (<1000 daltons) cannot elicit immune responses in native form, therefore, LMW sensitizers are found to be electrophiles that can covalently bind to proteins. For this reason, it is understood that the molecular initiating event common to all LMW sensitizers involves binding with proteins encountered in the epithelial layers. Molecules with the ability to bind proteins can generate hapten-protein complexes that are capable of eliciting immune responses. However, electrophilic reactivity is not specific enough to identify sensitizers (Enoch et al., 2009). Further, non-sensitizing chemicals may be transformed *in situ* into more reactive species by oxidative and/or metabolic processes. As a result, more complex NAMs may need to be used in concert with reactivity profiling to identify haptens as well as pre- and pro-haptens.

**FIGURE 1:**
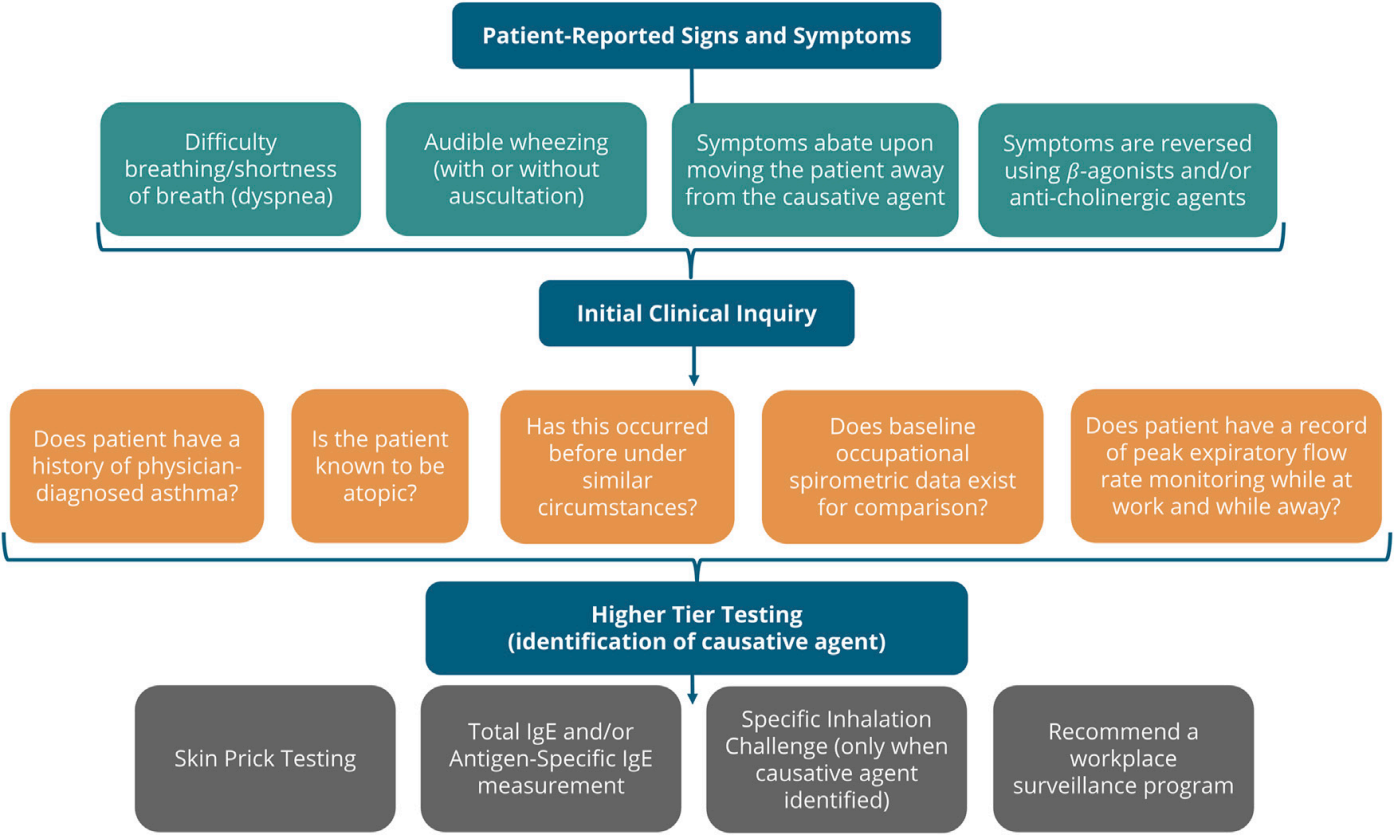
Clinical diagnosis of Allergic Asthma. The clinical inquiry following presentation of a patient suspected of chemical allergy is shown. If the symptoms occur following exposure in the workplace, the clinician may pursue the identification of any materials in the workplace suspected to be causative. If enough evidence can be collected, higher tier testing demonstrating an immune-mediated respiratory response is consistent with respiratory sensitization (Friedman-Jiménez et al., 2000; Beckett, 2008; Malo et al., 2015).

For our purposes, understanding clinical signs, symptoms and testing reported in the literature that can assist in the identification of occupational asthma cases that reflect respiratory sensitization by exposure to LMW chemicals was necessary to screen our “in litero” candidates. The relevant clinical diagnosis for this adverse outcome, asthma, is defined as a condition of variable airflow obstruction, often presenting with symptoms of cough, wheeze, shortness of breath and/or chest tightness (Beckett, 2008). The etiology of asthmatic responses may be the result of either an irritation-induced, non-immunologic mechanism or an immune-mediated adaptive response. A synopsis of a tiered clinical approach to evaluation of individuals presenting with signs and symptoms of asthma from exposure to an allergenic material is shown in Figure 1.

Identifying causative agents in occupational asthma is complicated by the likelihood of confounding exposures in the workplace. When occupational asthma is suspected, specific inhalation challenge (SIC) is often difficult due to the presence of confounding materials in the environment and, more importantly, the reality of risk to the patient. In allergic asthma, the condition develops as a result of two phases: induction (sensitization), with initial exposure to the causative agent, and elicitation (allergic response) upon re-challenge (Sayes, 2018). Controlled workplace challenge tests with a measurement of serial peak expiratory flow or spirometry, specifically, forced expiratory volume in the first second (FEV_1_), while in the workplace and away, can be helpful (Beckett, 1994). Due to the overlap in outward symptoms, a variety of methods are combined to distinguish irritant-induced versus immune-mediated responses (Sayes, 2018; Pemberton and Kimber, 2021).

**FIGURE 2:**
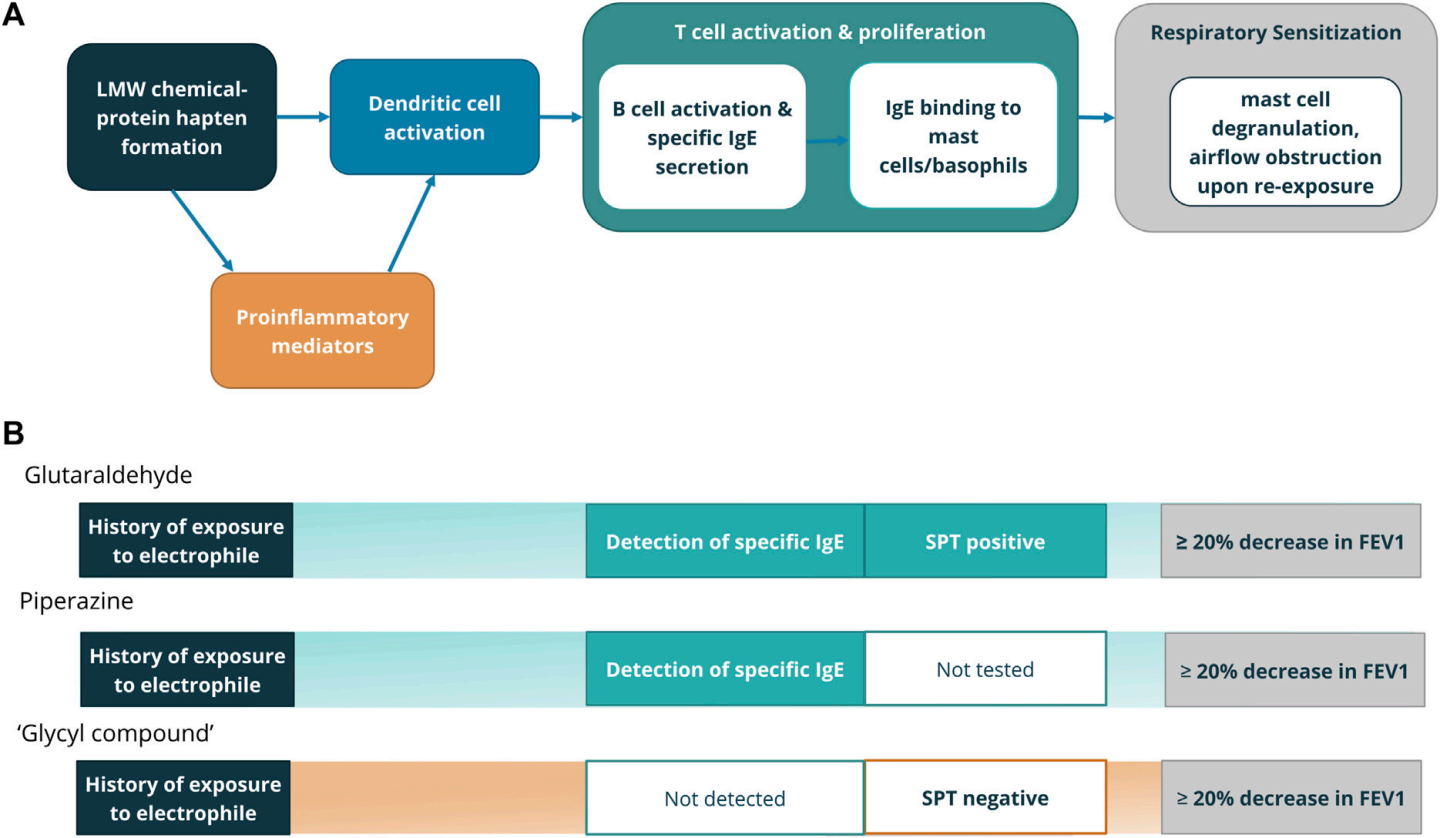
**(A)** Clinical diagnostic tests aligning with Key Events in the AOP for respiratory sensitization from LMW chemicals (Beckett, 2008; Sullivan et al., 2017). **(B)** Evidence in case reports that point to Key Events. Chemicals for which case reports included evidence of exposure, immune involvement either through direct detection of specific IgE or IgG, or indirectly through a skin prick test, as well as demonstrated airflow obstruction were determined to be clinical respiratory sensitizers. Representative chemicals from clinical respiratory sensitizer (glutaraldehyde, piperazine) and equivocal (‘glycyl compound’) categories are shown with clinical evidence leading to category decision.

With these considerations, we aligned the available clinical history, signs, and symptoms typically available in occupational asthma case reports within the framework of the AOP (Figure 2) to create key event based criteria to identify relevant diagnostic testing, such that only chemicals showing both a consistent history to identify a causative role in occupational asthma as well as conclusive T cell activation by the chemical or hapten-protein conjugate were identified as clinical respiratory sensitizers.

## 2 Methods

**FIGURE 3:**
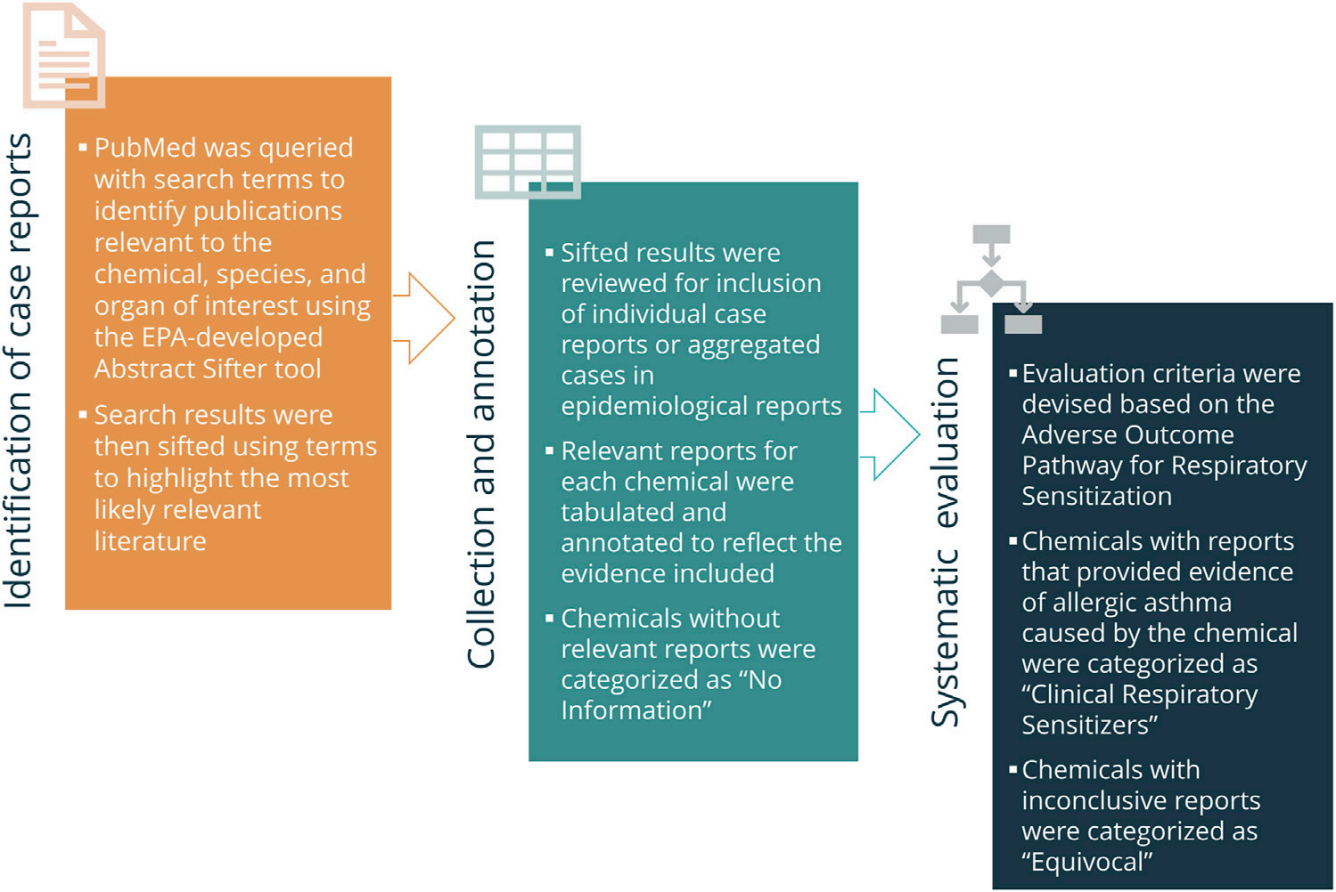
Generalized “In Litero” screening approach. In order to identify clinical literature, Abstract Sifter was used to collect and “sift” the most relevant results to the top. Reports referring to clinical cases of occupational asthma associated with each chemical were tabulated and annotated. Chemicals for which relevant reports could not be identified were categorized as “No Information” and excluded from evaluation. The reports for the remaining chemicals were categorized according to the established evaluation criteria as “Clinical Respiratory Sensitizers” or “Equivocal”.

A reference list of putative respiratory sensitizers was built with 118 chemicals identified primarily by a structure-based literature search to identify human clinical indication of respiratory sensitization (Enoch et al., 2012). In order to systematically curate this list, a phased approach was developed for mining the published literature for relevant reports and setting specific criteria for acceptability of the data into decision making, the key decision being whether the compound has been shown to cause respiratory sensitization from occupational or environmental exposures in clinical literature (Figure 3).

### 2.1 Primary Literature Search Using Abstract Sifter

The EPA-developed Abstract Sifter (version 5.5) literature review tool^2^ was used to maximize the chances of finding the right information for each chemical (Baker et al., 2017). Each of the chemicals were queried using the terms **chemical name AND (human OR clinical) AND (respiratory OR lung OR asthma)** to collect as much clinical literature referring to both the chemical and the organ of interest in the abstract. If limited search results appeared, the qualifier terms were removed, and the query was re-run with the **chemical name** alone. Alternatively, if results exceeded the maximum processing limit of the tool, an additional qualifier of AND **(asthma OR allergy)** was used along with the initial search terms and the query was re-run. To further filter the results to most relevant reports, sifter terms such as **Immunoglobulin E**, **bronchial challenge**, and **sensitize** were used to “sift” the most promising literature to the top of the list. A separate Abstract Sifter file was prepared for each chemical, and the sifted results were taken into consideration for data analysis and application of decision-making criteria. Searches were repeated with one or more chemical synonyms where necessary to retrieve relevant literature.

### 2.2 Key Factors Used When Considering Published Reports

#### 2.2.1 History of Exposure to the Chemical

Our first consideration was whether the patient had been previously exposed to the compound in an occupational or other environmental context, and onset of symptoms including asthmatic manifestation and/or rhinitis followed exposure or recurred with re-exposure. Wherever available, evidence of direct and precise association of the exposure to the compound resulting in symptoms and cessation of symptoms upon elimination of exposure was also considered.

#### 2.2.2 Evidence of Specificity of Symptoms

While considering data from bronchial challenge tests, it was noted whether the bronchoprovocation test was to methacholine or histamine, non-specific, or if a specific bronchial challenge using the compound in question was performed. Non-specific inhalation challenges contributed towards evidence of specificity when paired with negative controls to eliminate other ingredients in the sensitizing challenge material.

#### 2.2.3 Evidence of Specific IgE And/Or IgG Immune-Mediated Mechanism

Detection of chemical specific IgG and/or IgE, either directly, through radioallergosorbent test (RAST) or enzyme-linked immunosorbent assay (ELISA), or indirectly, by a wheal and flare reaction in response to a skin prick test (SPT) or intra-dermal test (IDT), was noted. Wherever data was available, additional points such as whether any controls were tested and if the antigen used was conjugated or unconjugated were also noted. While evidence of specific immune involvement was required for the category of clinical respiratory sensitizer, a positive response to a mixture was considered where it was paired with negative controls to eliminate other ingredients in the mixture.

#### 2.2.4 Evidence of Other Mechanisms

Where available, other mechanistic data, such as allergen triggered basophil activation and histamine release and measurements of T-helper 2 cytokines were also included as part of the weight of evidence (Shamji et al., 2017). These mechanisms were considered suggestive of respiratory sensitization, although not necessary for categorization.

#### 2.2.5 Presence of Any Confounding Exposures

Any other chemical exposure that could have caused the sensitization, or onset of symptoms, thereby confounding the association of the queried chemical with respiratory sensitization, was a key consideration. If positive but nonspecific test results (*e.g.* bronchial challenge, skin prick test) were reported, whether negative controls to eliminate confounding exposures were included was noted.

### 2.3 Applying Defined Criteria for Classification of Clinical Respiratory Sensitizers

Incorporating considerations informed by both the adverse outcome pathway and the clinical diagnostic process, we developed the following criteria to identify chemicals as clinical respiratory sensitizers:

#### 2.3.1 No Information

There was not sufficient information to evaluate the compound, either:

a Information on the chemical was absent in the literature
b Available literature on the chemical was not relevant to human respiratory symptoms

#### 2.3.2 Equivocal

There is clinical evidence of respiratory symptoms after exposure, but available evidence does not conclusively demonstrate sensitization because either:

a There is no evidence of immune-mediated response, from the evidence and set data acceptability criteria related to specific IgE and/or IgG, to distinguish respiratory sensitization from respiratory irritation
b There is conflicting evidence of immune-mediated response or confounding exposure to other chemicals

#### 2.3.3 Clinical Respiratory Sensitizers

There is significant clinical evidence that the compound has caused respiratory sensitization in at least one individual, as defined by one of the following scenarios:

a There is patient history of exposure with positive specific bronchial challenge, combined with evidence of specific IgE and/or IgG immune-mediated response as determined by RAST/ELISA/SPT/IDT, upon exposure to the compound
b There is notable patient history of exposure with positive non-specific bronchial challenge or evidence of direct and precise association of the exposure to the compound resulting in symptoms, combined with evidence of IgE and/or IgG immune-mediated response, paired with negative controls to eliminate confounding exposures

Led by the key considerations and classification criteria, each of the 118 compounds, were classed into one of the three above groups by two of three evaluators. After completing the classification of compounds, an internal peer review was conducted to review each conflicting decision in accordance with the key factors and classification criteria among all three evaluators.

For all compounds classified as clinical respiratory sensitizers, the total number of patients identified meeting the criteria in the available literature was considered to indicate whether we observed a high (N > 10) or low (N ≤ 10) incidence of reports. For this definition, we considered each patient with consistent and conclusive case of allergic asthma from low molecular weight chemicals to be an individual report.

### 2.4 Additional Chemical Review

From the first review, chemicals that were classified as clinical respiratory sensitizers were used to generate a list of additional compounds that may have clinical data on respiratory sensitization. Using the Medical Subject Headings (MeSH) chemical terms for these chemicals and including only articles where the chemical was annotated as major topic, the literature database (EPALitDB) of MeSH annotations for disease terms was queried and the diseases related to respiratory sensitization were identified (Baker and Hemminger, 2010; Judson et al., 2019). The results showed that the chemicals co-occurred with at least one of these disease terms: Asthma, Bronchial Hyperreactivity, or Respiratory Hypersensitivity. This set of three disease terms was used to query the full chemical-disease database to find any other chemical associated with at least one of the three terms. Once again, only chemicals annotated as major topics were included. A filter was applied to remove non-organic chemicals. To expand the understanding of these chemicals, co-occurrences of these chemicals with other terms known for association with the mechanism of respiratory sensitization, e.g., Protein Binding, Antibody Formation, Immunoglobulin E, and Pulmonary Alveoli were assessed. All the chemical and co-annotation data was collected with the article information for browsing and evaluation. Of these additional materials, out of scope materials including mixtures, proteins, and high molecular weight polymers were excluded. For the remainder, the collected literature was evaluated according to the same classification criteria as the first set. Additional literature searches were not performed for the second set of materials.

### 2.5 Assignment of Market/Occupational Sector

Following classification, the major market/occupational sector for use of all chemicals included in the analysis was collected from public databases using name and CAS number queries (Dionisio et al., 2018; Kim et al., 2020). Broad sectors were used to ensure appropriate categorization into either industrial, pharmaceutical and medical, biocide, or food and cosmetics. This information was compared with the final classification for all compounds to evaluate the relative representation of chemicals from different occupations within our analysis.

### 2.6 Protein Binding Profiling

To characterize the protein binding mechanism of the chemicals in our dataset, the OECD QSAR Toolbox v4.4.1 was utilized. The QSAR Toolbox was developed by the OECD in collaboration with the European Chemical Agency (ECHA) as a standalone software application to assess the potential hazards of substances available for free download^3^. Several profilers within the QSAR Toolbox have been designed for identifying specific properties, including protein-binding profilers based on the OASIS algorithm and oxidation and metabolic activation. These profilers were used together to identify the mechanism(s) of protein binding for all chemicals analyzed. Chemicals were identified in the QSAR Toolbox by name and/or CAS number. Chemicals which had no binding alert for the parent molecule but had an alert for at least one metabolite or oxidation product were listed as having a “Pre-/Pro-hapten alert”.

## 3 Results

### 3.1 Identified Clinical Respiratory Sensitizers

**FIGURE 4:**
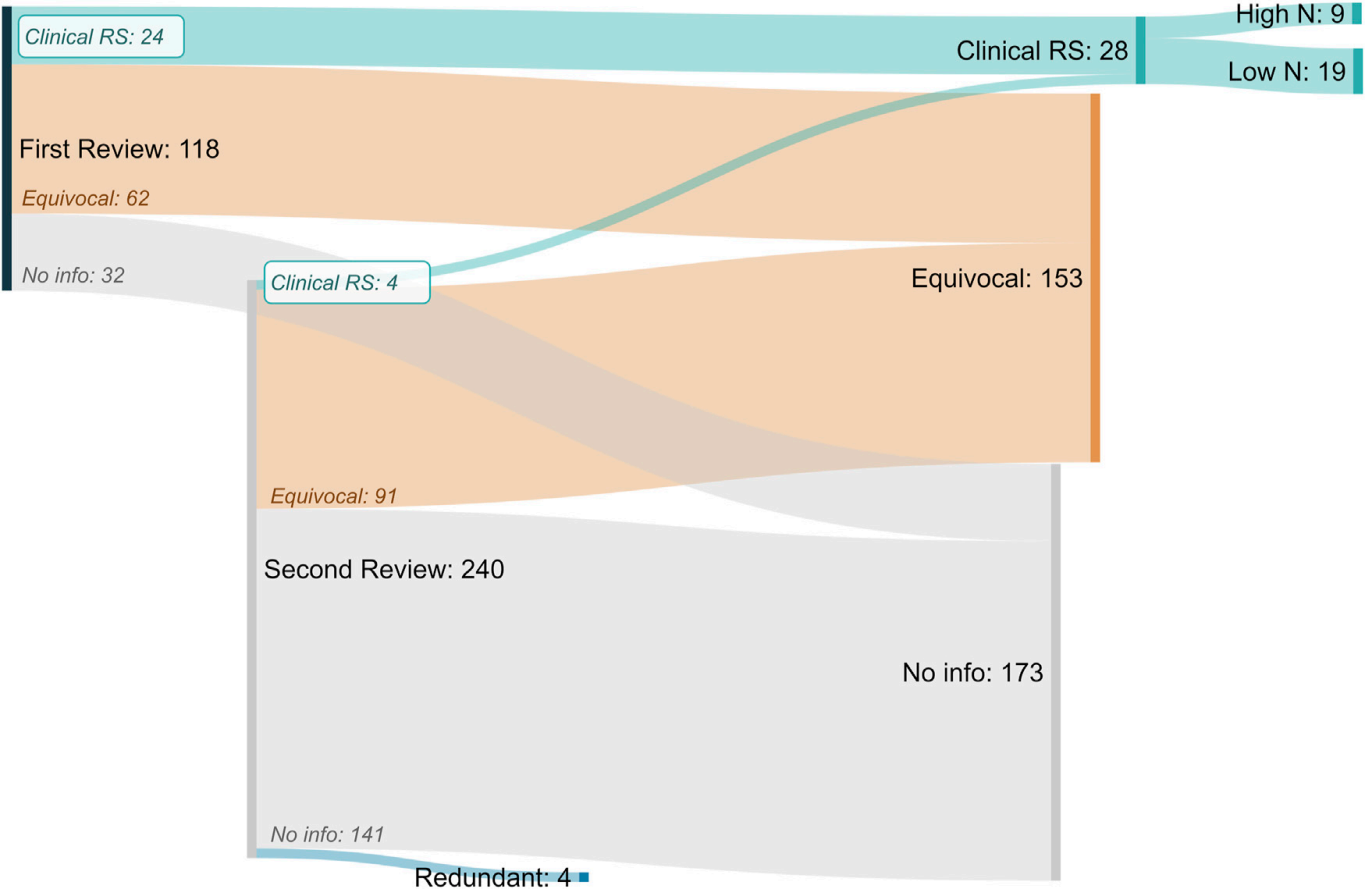
Sankey Diagram of “in litero” screening results for all putative respiratory sensitizers and related compounds. From the original review of 118 putative sensitizers, 24 clinical respiratory sensitizers (Clinical RS) were identified. An additional automated search for similarly reported chemicals identified 240 chemicals, resulting in the identification of 4 more clinical respiratory sensitizers. For nine of the total 28 clinical respiratory sensitizers identified, we found case reports demonstrating respiratory sensitization in greater than ten total patients (High N). For the remaining 19, at least one case of clinical respiratory sensitization was identified (Low N). Inconclusive (Equivocal) evidence suggestive of clinical respiratory sensitization was found for an additional 153 chemicals.

In total, 354 chemicals were investigated in this effort resulting in the identification of 28 total clinical respiratory sensitizers (Figure 4). The list of identified clinical respiratory sensitizers is given in Table 1 with references to the evaluated case reports and other clinical literature leading to the classification. A high incidence (>10) of asthma patients (termed “High N”) with consistent case history and specific IgE or IgG was found for 9 the 28 identified clinical respiratory sensitizers. For several of the low incidence (Low N, N ≤ 10) sensitizers, a single case of respiratory sensitization was reported with the chemical identified as the specific causative agent. In our approach, a single, well-documented case was considered sufficient to classify a compound as a clinical respiratory sensitizer.

**TABLE 1:** Identified Clinical Respiratory Sensitizers. A total of 28 clinical respiratory sensitizers were identified in our approach. Low N: 10 or fewer cases; High N: greater than 10 cases. †Two clinical skin sensitizers previously identified by human predictive patch tests (OECD, 2021) were identified as clinical respiratory sensitizers in our approach.

Chemical name	CAS#	Incidence	References
2-(1H-benzotriazole-1-yl)-1,1,3,3-tetramethyluronium tetrafluoroborate	125700–67–6	Low N	Miralles et al. (2003)
2-(1H-benzotriazole-1-yl)-1,1,3,3-tetramethyluronium hexafluorophosphate	94790–37–1	Low N	Miralles et al. (2003)
2,4-dichloro-5-chlorsulfonyl-benzoic acid	3740–18–9	Low N	Klusáčková et al. (2007)
7-aminocephalosporanic acid	957–68–6	Low N	Park et al. (2004)
Ammonium hexachloroplatinate	16919–58–7	High N	Merget et al. (1994), Newman Taylor et al. (1999), Merget et al. (2001)
Ammonium persulfate	7727–54–0	Low N	Hagemeyer et al. (2015)
Ampicillin	69–53–4	Low N	Wüthrich and Hartmann (1982)
Carmine	1328–60–5	Low N	Cox and Ebo (2012)
Cefadroxil	50370–12–2	Low N	Choi et al. (2002)
Cefteram Pivoxil	82547–81–7	Low N	Suh et al. (2003)
Chloramine-T (Sodium p-toluenesulfonylchloramide)	127–65–1	Low N	Helaskoski et al. (2015)
Formaldehyde†	50–00–0	Low N	Baba et al. (2000)
Glutaraldehyde†	111–30–8	High N	Curran et al. (1996), Di Stefano et al. (1999), Baba et al. (2000)
Hexahydrophthalic anhydride (HHPA)	85–42–7	High N	Moller et al. (1985); Grammer et al. (1994)
Hexamethylene diisocyanate (HDI)	822–06–0	Low N	Wass and Belin (1989)
Menthol	1490–04–6	Low N	dos Santos et al. (2001)
Methylene diphenyl diisocyanate (MDI)	101–68–8	Low N	Rudbeck and Omland (2006)
Methyl tetrahydrophthalic anhydride (MTHPA)	11070–44–3	High N	Nielsen et al. (1989), Nielsen et al. (1994)
Pauli’s reagent (4-diazobenzenesulfonic acid)	305–80–6	Low N	Evans and Seaton (1979)
Phenylglycine acid chloride	39878–87–0	Low N	Kammermeyer and Mathews (1973)
Phthalic anhydride (PA)	85–44–9	High N	Wernfors et al. (1986); Nielsen et al. (1988)
Piperacillin	61477–96–1	Low N	Moscato et al. (1995)
Piperazine	110–85–0	High N	Hagmar et al. (1982), Hagmar and Welinder (1986), Quirce et al. (2006)
Plicatic acid	16462–65–0	High N	Chan-Yeung (1982), Cartier et al. (1986), Vedal et al. (1986), Paggiaro and Chan Yeung (1987)
Potassium dichromate	7778–50–9	Low N	Olaguibel and Basomba (1989), Bright et al. (1997), Fernández-Nieto et al. (2006)
Thiamphenicol	15318–45–3	Low N	Ye et al. (2006)
Toluene diisocyanate (TDI)	26471–62–5	High N	Weill et al. (1981), Duan (1989), Park et al. (2001)
Trimellitic anhydride (TMA)	552–30–7	High N	Zeiss et al. (1977); Zeiss et al. (1982); Zeiss et al. (1990)

**FIGURE 5:**
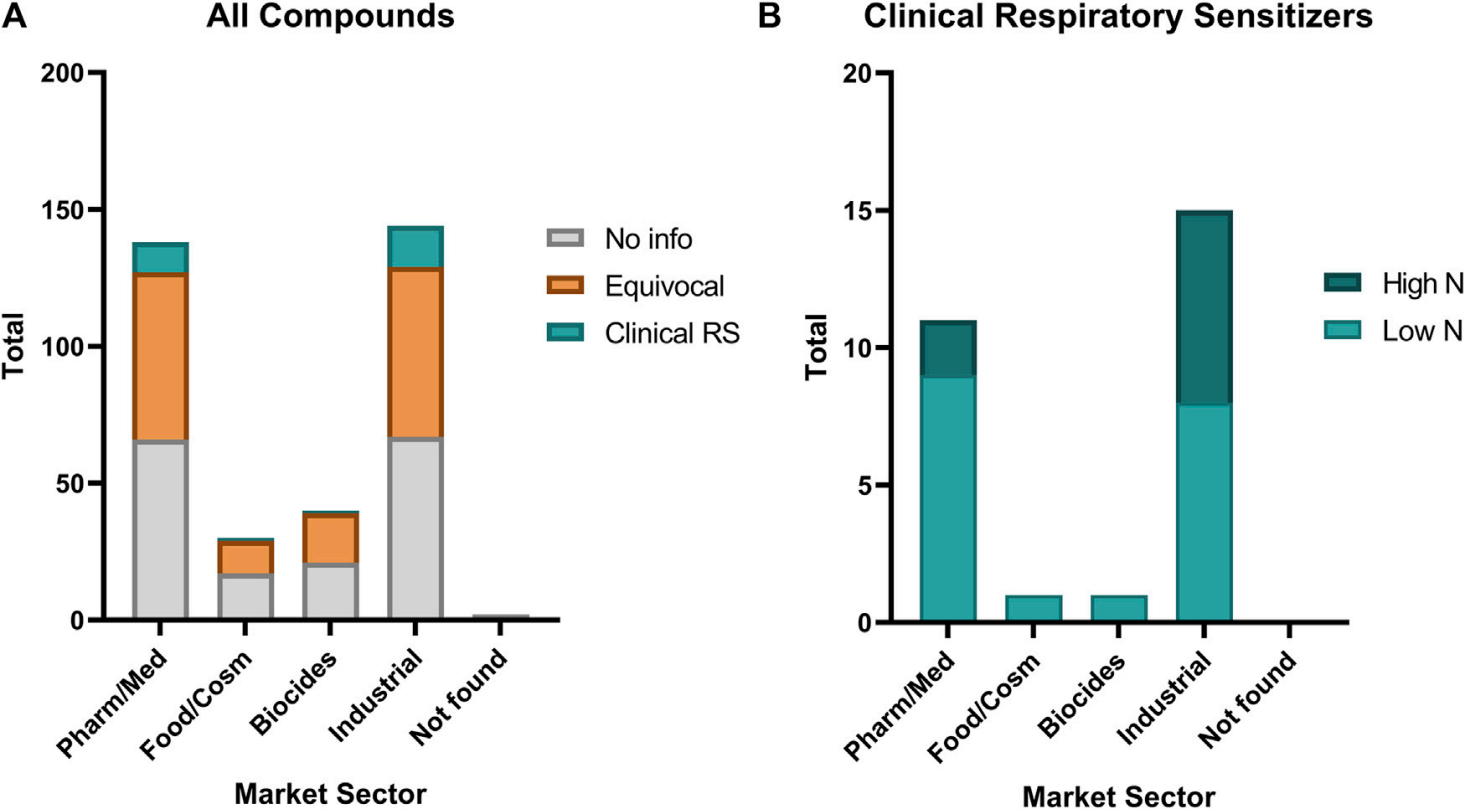
Relative distribution of occupational sector of **(A)** all evaluated chemicals compared with **(B)** the identified clinical respiratory sensitizers (Clinical RS). A small percentage (2—10%) of chemicals in each market sector represented in our investigation were found to be clinical respiratory sensitizers. Low N: 10 or fewer cases; High N: greater than 10 cases. Pharm/Med: pharmaceutical and medical. Food/Cosm: food and cosmetic. Biocides: insecticide, fungicide, antimicrobials, and other pesticides. Industrial: industrial materials, especially polymerizing agents. Not found: a market sector of use could not be identified for this compound in available public databases.

The identified clinical respiratory sensitizers represented primarily chemicals from industrial and pharmaceutical or medical uses (Figure 5). One food and cosmetic ingredient (carmine) and one biocide (Chloramine-T) were identified. Comparing the relative distribution of classification as “No information,” “Equivocal,” and “Clinical Respiratory Sensitizer” among all the chemicals investigated, we find that our chemical set is fairly representative of health hazard risks from electrophiles in occupational settings, and sensitizers are found in approximately the same proportion (≤10%) of chemicals investigated from each market sector. No significant association is apparent between any particular market/occupational sector and the frequency of identified clinical respiratory sensitizers. A slight trend favoring a higher incidence of reports from industrial settings is observed, which may be reflective of the historical exposures to classical industrial respiratory sensitizers such as anhydrides and isocyanates.

### 3.2 Protein Binding Profiles of Clinical Respiratory Sensitizers

**FIGURE 6:**
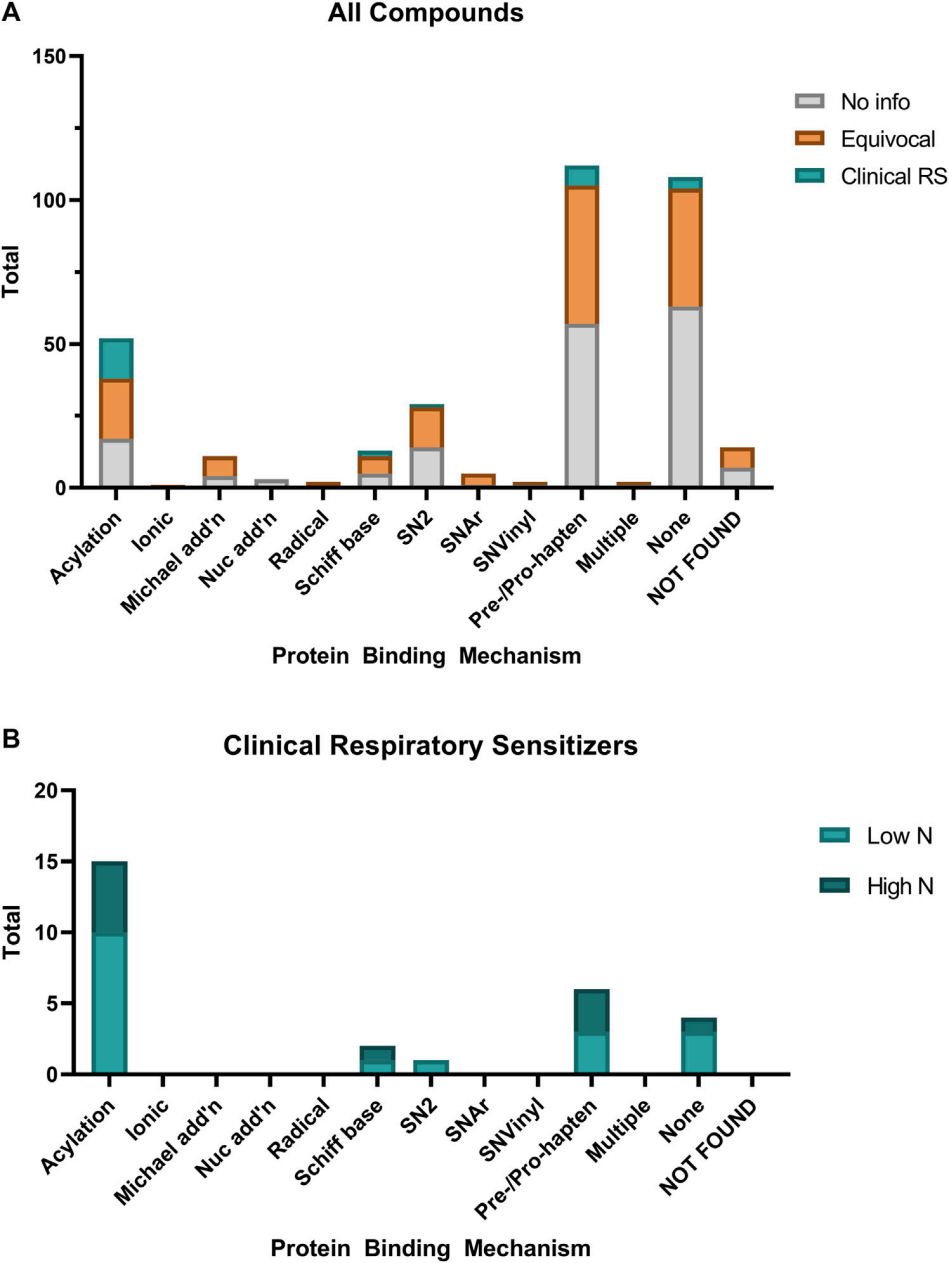
Relative distribution of protein binding mechanisms of **(A)** all evaluated chemicals compared with **(B)** the identified clinical respiratory sensitizers. Protein binding mechanisms were predicted using the OECD QSAR Toolbox. Chemicals which had no protein binding alerts except when predicted metabolites or oxidation products were profiled are included as “Pre/Pro-hapten.”

In order to explore what our dataset of respiratory sensitizers may show about the molecular initiating event of respiratory sensitizers, we used the QSAR Toolbox (v. 4.4.1, OECD) to profile the protein binding mechanism(s) of each of the 354 chemicals within our investigation set (Figure 6). Many (108) chemicals had no protein binding alerts, while a similar number 112) were found to only have “Pre- and Pro-Hapten” protein binding alerts, *i.e.* alerts only for predicted metabolites or auto-oxidation products. Fourteen chemicals could not be found within the QSAR Toolbox database. Most notably, a significant (*p* < 0.0001, Fisher’s Exact Test) association was found between protein binding by acylation relative to other mechanisms, and the designation as a clinical respiratory sensitizer within our dataset. This is found to be in contrast to dermal sensitizers, which are most often associated with Michael addition, nucleophilic substitution, and Schiff base formation (Dearden et al., 2015; OECD, 2021). Both the protein binding alerts and occupational/market sectors are presented in Supplementary Table S1 for the identified clinical respiratory sensitizers.

## 4 Discussion

### 4.1 “In Litero” Approach to Identify Clinical Respiratory Sensitizers

The use of Abstract Sifter greatly simplified the task of collecting and ranking the relevance of literature for both well-studied chemicals that retrieve tens of thousands of publications as well as uncommon chemicals with very few published references, whether related to clinical literature or not. An example of the Abstract Sifter output is available as Supplementary Table S2. On occasion, results were too numerous to be processed by the tool and additional qualifiers were necessary to refine results. For chemicals where few references were retrieved, a manual literature search was conducted to verify a lack of relevant literature. In a few cases, additional references were noted by this manual search, but no additional sensitizers were identified.

Applying the established criteria to the collected literature was not always straightforward. Occupational exposures are often concurrent, with multiple potential causative agents in the workplace or material being handled, especially for industrial and pharmaceutical manufacturing. Cross-reactivity and co-sensitization of individuals to multiple chemicals from the workplace was a significant contributor to the inability to reliably attribute a case of sensitization to a specific chemical. This is particularly true in the case of reactive dyes, where there are many reports of clinical respiratory sensitization, but workers are typically exposed to two or more dyes and become sensitized to multiple dyes after repeated exposures. In these cases, although one or more dyes are demonstrated to be capable of causing respiratory sensitization, our approach cannot isolate any single dye as being conclusively causative of the outcome.

The presence of confounders with a lack of proper controls was a common reason for classification as “Equivocal”. This classification was given to 153 chemicals, representing nearly half of the chemicals investigated (Figure 4). In most of these cases, a lack of confirmation of T-cell activation in the form of specific IgE or IgG led to the inability to classify the chemical as a sensitizer. Often, no such testing was conducted. Importantly, for respiratory sensitization, a negative result is not suggestive of a lack of sensitization, as the role of IgE and IgG are not well-characterized. However, there are no other clinical tests to confirm immune involvement and thus discriminate between respiratory irritant and sensitizer effects, so this was a necessary requirement to identify clinical respiratory sensitizers.

As might be expected, for many chemicals there were no clinical reports to evaluate, and this proportion was larger in the second phase of review. The additional chemicals identified by this additional search were mostly (141/240) low information chemicals. However, this phase was helpful for identifying literature referring to chemical synonyms as well as sensitizers not represented in the original structure-based set. Four of the chemicals evaluated in this phase were found to be synonymous with chemicals in the original set of 118 chemicals. Four additional clinical respiratory sensitizers were identified according to our previously established criteria from this additional search. One chemical identified in the second phase contained the same organic formula as a previously identified clinical respiratory sensitizer, paired with a different inorganic counterion.

Several approaches for identifying a reference list of respiratory sensitizers using literature reviews have been described. A sizable number (78) of causative agents for allergic asthma with varying evidence strengths was described, however, most of these identified causes were mixtures or foods including proteins, and very few of these represented individual LMW chemicals (Baur, 2013). Our analysis is mostly in agreement with a recent review that identified seven LMW respiratory sensitizers for which compelling evidence exists (Sadekar et al., 2022). All seven of these were identified as clinical respiratory sensitizers in our analysis, despite differing approaches and cited literature used in the determination. Sadekar and colleagues evaluated evidence from public and non-public databases for a set of 97 LMW chemicals identified as potential respiratory sensitizers, sorting chemicals into categories that reflected both the quality and quantity of clinical reports. In contrast, although we used similar clinical criteria, our “in litero” approach focused on the quality of clinical evidence, although we annotated identified sensitizers to reflect the quantity of cases reported that met our clinical criteria.

To the authors knowledge, a comparable literature review approach has not been conducted for skin sensitizers, for which human predictive patch test (HPPT) data is common. A comprehensive review of HPPT data was recently conducted (OECD, 2021), however, there is very little overlap between those chemicals and our identified clinical respiratory sensitizers, likely at least in part due to acute toxicity. However, two of the identified clinical respiratory sensitizers, glutaraldehyde and formaldehyde, were identified as clinical skin sensitizers. These are noted in Table 1.

### 4.2 Advantages and Limitations of “in Litero” Screening Approach

The primary advantage of our approach was the collection and curation of case reports and related epidemiological literature with demonstrative evidence of occupational asthma from LMW chemical exposures. The list of 28 clinical respiratory sensitizers identified here can be used with high confidence with respect to human relevance within a weight-of-evidence approach to defining respiratory sensitizers. Retrospective clinical literature analyses are an important tool to synthesize existing clinical data when prospective clinical testing is not feasible. As we improve our understanding of the poor reliability and human relevance of legacy *in vivo* data, these analyses will need to take precedence when validating NAMs for the purpose of protecting human health. A recent review highlighted the issue of respiratory sensitization as an opportunity for clinicians and toxicologists to work together to improve our understanding of causative agents in allergic asthma (Pemberton and Kimber, 2021). This approach represents an important first step toward realizing this opportunity, establishing a bedside-to-bench framework of relying on clinical epidemiology of the AO to inform on the utility of available NAMs addressing the molecular initiating eventand downstream key events in the etiology of respiratory sensitization.

This approach relied on automated search methods which are subject to common limitations, including different spelling conventions (*e.g.* sensitization, sensitisation) and inconsistent chemical nomenclature. This was especially apparent when reviewing the accumulated literature for the additional compounds. As described, at least four of these “new” chemicals were already represented in our original chemical list (Figure 4) but were identified again due to inconsistent nomenclature. Additionally, one of our clinical respiratory sensitizers, methylene diphenyl diisocyanate (MDI), was assigned an “Equivocal” status in the first round of review as we were unable to identify any literature demonstrating specific immune involvement using searches based on the chemical name. However, in the additional chemical review we identified a case report in which allergic asthma with specific IgE response to MDI was detected, but MDI was mistakenly referred to as “methylene diisocyanate,” allowing us to conclude that MDI is a clinical respiratory sensitizer (Rudbeck and Omland, 2006). This reinforces the value of the additional chemical review, particularly for clinical literature where chemical nomenclature may be less standardized.

With a retrospective analysis, we could not control for the frequency of exposures, likelihood of implementing/adhering to personal protective equipment (PPE), or any other socioeconomic factors influencing the prevalence of case reports. Further, within those documented occupational asthma cases reviewed, we could not control for the likelihood of testing or methods used when testing for specific IgE and/or IgG. For these reasons, we did not exclude chemicals from our list of identified sensitizers based on relative frequency or incidence of reports. This consideration is a critical factor in the interpretation of our collected evidence and our defined criteria. There are many reasonable scenarios that could lead to a true respiratory sensitizer not being identified retrospectively as a clinical respiratory sensitizer, not least of which is high attention to occupational safety and appropriate use of PPE. In this sense, the higher incidence of clinical respiratory sensitizers identified in industrial settings relative to pharmaceutical and medical occupations may reflect historical influence rather than relative occupational risk.

Another specific limitation to our approach is the inability to account for potency or duration of exposures. Identification of causative agents in occupational asthma does not consistently include estimates of inhaled exposures, either frequent or incidental. Further, in a retrospective analysis, it is not known whether a “weak” response is attributable to the causative material, or the conditions of exposure, or differences in individual susceptibility. We therefore did not include potency or duration in the consideration of our qualitative classification. Additionally, we found that many clinical indicators of airway inflammation were rarely reported, including rhinitis, induced sputum counts, exhaled nitric oxide, and Th2-specific cytokine profiling. While clinicians are, fittingly, concerned first with protecting the patient, these results demonstrate the practical utility in developing standardized testing and reporting strategies between toxicologists and clinicians to help elucidate mechanisms using what information can be gathered noninvasively from incidental exposures.

It is important to note that our approach was designed for a specific purpose: to identify a list of respiratory sensitizers with the highest confidence with respect to human relevance for the purpose of incorporating this evidence stream into a weight-of-evidence approach. A full weight-of-evidence approach will ultimately require a defined set of both positive and negative reference chemicals for the development of clinically relevant hazard characterization approaches. Our approach did not attempt to review literature for the purpose of identifying non-sensitizers. Such an approach could be a valuable addition to a weight-of-evidence analysis but would require an entirely different literature collection and evaluation method. However, as non-sensitizing materials are much more common than sensitizers, a significant “in litero” screen may not ultimately be necessary to identify a sufficient number of high-confidence negatives.

Finally, our results strongly suggest that the biological mechanism of respiratory sensitizers and skin sensitizers is distinct at the molecular initiating event level. This adds to the expanding body of evidence that shows that dermal and respiratory sensitization are likely to be discrete pathways. The identification of this set of clinical respiratory sensitizers is a crucial step towards devising NAMs that can assess respiratory and dermal sensitization independently. New understanding of the distinctions between these two pathways may lead to revisions of one or more key events shared by both AOPs.

## Data Availability

The original contributions presented in the study are included in the article/Supplementary Material, further inquiries can be directed to the corresponding author.

## Footnotes

1
https://aopwiki.org/aops/39

2
https://github.com/USEPA/CompTox-Chemistry-Dashboard-Abstract-Sifter

3
http://toolbox.oasis-lmc.org
