# Supplementary material for: “In Litero” Screening: Retrospective Evaluation of Clinical Evidence to Establish a Reference List of Human Chemical Respiratory Sensitizers
Source: Front Toxicol. 2022 Jul 15;4:916370. doi: 10.3389/ftox.2022.916370 (PMC9335368; doi:10.3389/ftox.2022.916370)
Supplement: Supplementary file 2 [file Table1.DOCX]

Supplementary Material

# Supplementary Table 1: Market/occupational sector and protein binding mechanisms of the identified clinical respiratory sensitizers.

Protein binding mechanisms were predicted using the OECD QSAR Toolbox. Chemicals which had no protein binding profiles except when predicted metabolites or oxidation products were profiled are included as “Pre/Pro-hapten.”

| CHEMICAL NAME | CAS# | MARKET sector | PROTEIN BINDING Alert |
| --- | --- | --- | --- |
| 2-(1H-benzotriazole-1-yl)-1,1,3,3-tetramethyluronium tetrafluoroborate | 125700-67-6 | Industrial | Pre/Pro-hapten |
| 2-(1H-benzotriazole-1-yl)-1,1,3,3-tetramethyluronium hexafluorophosphate | 94790-37-1 | Industrial | Pre/Pro-hapten |
| 2,4-dichloro-5-chlorsulfonyl-benzoic acid | 3740-18-9 | Medical | Acylation alert |
| 7-aminocephalosporanic acid | 957-68-6 | Pharmaceutical | Acylation alert |
| Ammonium hexachloroplatinate | 16919-58-7 | Industrial | No alert |
| Ammonium persulfate | 7727-54-0 | Industrial | No alert |
| Ampicillin | 69-53-4 | Pharmaceutical | Acylation alert |
| Carmine | 1328-60-5 | Food | Pre/Pro-hapten |
| Cefadroxil | 50370-12-2 | Pharmaceutical | Acylation alert |
| Cefteram Pivoxil | 82547-81-7 | Pharmaceutical | Acylation alert |
| Chloramine-T (Sodium p-toluenesulfonylchloramide) | 127-65-1 | Biocide | Pre/Pro-hapten |
| Formaldehyde† | 50-00-0 | Industrial | Schiff base alert |
| Glutaraldehyde† | 111-30-8 | Industrial | Schiff base alert |
| Hexahydrophthalic anhydride (HHPA) | 85-42-7 | Industrial | Acylation alert |
| Hexamethylene diisocyanate (HDI) | 822-06-0 | Industrial | Acylation alert |
| Menthol | 1490-04-6 | Pharmaceutical | Pre/Pro-hapten |
| Methylene diphenyl diisocyanate (MDI) | 101-68-8 | Industrial | Acylation alert |
| Methyl tetrahydrophthalic anhydride (MTHPA) | 11070-44-3 | Industrial | Acylation alert |
| Pauli's reagent (4-diazobenzenesulfonic acid) | 305-80-6 | Industrial | No alert |
| Phenylglycine acid chloride | 39878-87-0 | Pharmaceutical | Acylation alert |
| Phthalic anhydride (PA) | 85-44-9 | Industrial | Acylation alert |
| Piperacillin | 61477-96-1 | Pharmaceutical | Acylation alert |
| Piperazine | 110-85-0 | Pharmaceutical | Pre/Pro-hapten |
| Plicatic acid | 16462-65-0 | Industrial | Pre/Pro-hapten |
| Potassium dichromate | 7778-50-9 | Industrial | No alert |
| Thiamphenicol | 15318-45-3 | Pharmaceutical | S_N_2 alert |
| Toluene diisocyanate (TDI) | 26471-62-5 | Industrial | Acylation alert |
| Trimellitic anhydride (TMA) | 552-30-7 | Industrial | Acylation alert |
